# Durotaxis and negative durotaxis: where should cells go?

**DOI:** 10.1038/s42003-023-05554-y

**Published:** 2023-11-16

**Authors:** Congcong Ji, Yuxing Huang

**Affiliations:** 1 Key laboratory of Carcinogenesis and Translational Research (Ministry of Education/Beijing), Department of Gastrointestinal Oncology, Peking University Cancer Hospital & Institute, Beijing, 100142 China; 2 Center for Precision Medicine Multi-Omics Research, Peking University Health Science Center, Peking University, Beijing, 100191 China

**Keywords:** Mesenchymal migration, Collective cell migration

## Abstract

Durotaxis and negative durotaxis are processes in which cell migration is directed by extracellular stiffness. Durotaxis is the tendency of cells to migrate toward stiffer areas, while negative durotaxis occurs when cells migrate toward regions with lower stiffness. The mechanisms of both processes are not yet fully understood. Additionally, the connection between durotaxis and negative durotaxis remains unclear. In this review, we compare the mechanisms underlying durotaxis and negative durotaxis, summarize the basic principles of both, discuss the possible reasons why some cell types exhibit durotaxis while others exhibit negative durotaxis, propose mechanisms of switching between these processes, and emphasize the challenges in the investigation of durotaxis and negative durotaxis.

## Abstract (web summary)

This mini review highlights and compares the principles of durotaxis and negative durotaxis as part of cell migratory processes.

## Introduction

Cell migration plays important roles in development, homeostasis, immunity, and disease^1–5^. Cell migration strategies include mesenchymal cell migration, amoeboid migration, and collective cell migration. Mesenchymal cell migration is the best-studied process. Typically, mesenchymal cell migration involves four steps: the protrusion of the leading edge, the formation of initial adhesion, the contraction of myosin motors, and the disassembly of adhesion in the rear^6^. However, it should be noted that the concept of four-step migration does not apply to cells that undergo amoeboid migration or collective cell migration. Amoeboid migration is characterized by low adhesion and fast movement, and it is utilized by various cell types, including immune cells and single-cell social amoebas^7^. The protrusions of amoeboid migration can range from actin-based lamellipodia and filopodia to myosin-based spherical blebs^8^. A recent study reported that amoeboid cells, such as T cells, neutrophils, and Dictyostelium, exhibit durotaxis^9^. The microenvironment contains various stimuli, such as chemokines, light, and electrons, which induce directed cell migration, including chemotaxis, phototaxis, and electrotaxis^10–12^. In this context, how do cells migrate toward or away from these stimuli? Directed cell migration is reported to involve three steps^13^. First, cells sense stimuli by using extracellular or intracellular receptors. Second, cells transduce extracellular signals into intracellular signals and generate cell polarity under the regulation of the Cdc42-Par-aPKC pathway and small GTPases such as Rac1 and RhoA^14–16^. Finally, cells migrate following the basic principles of cell migration.

**Fig. 1:**
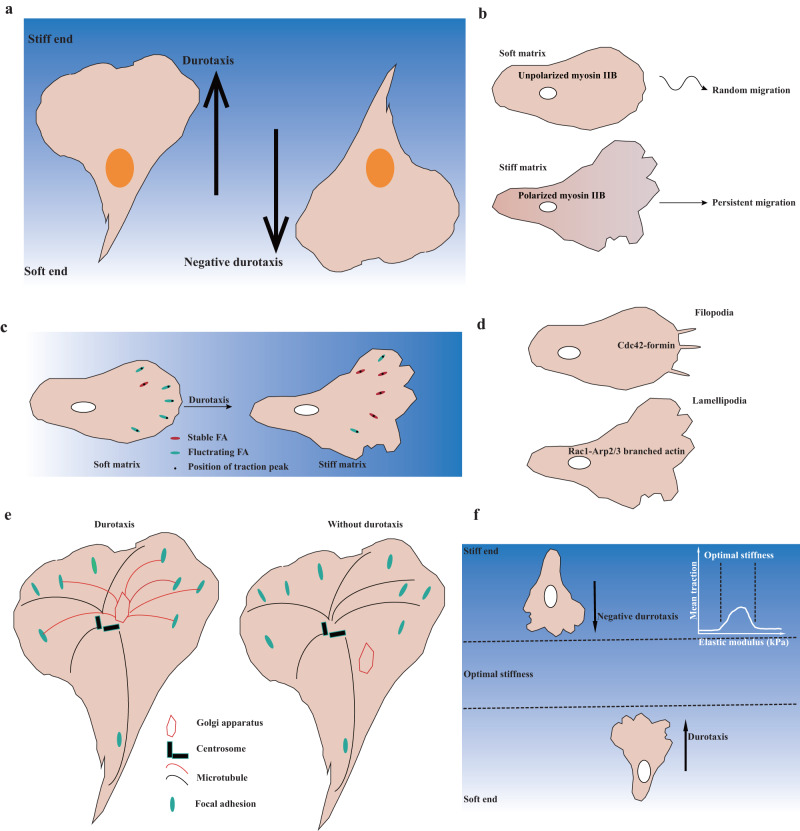
Mechanisms of durotaxis and negative durotaxis. **a** Cell migration is directed by extracellular matrix stiffness. Left: the ability of cells to migrate from the soft end to the stiff end of the extracellular matrix is called durotaxis. Right: the ability of cells to migrate from the stiff end to the soft end of the extracellular matrix is called negative durotaxis. **b** Myosin IIB is unpolarized in cells on a soft matrix, both in 2D and 3D. However, it becomes repolarized as the cells crawl from a soft to a stiff matrix. Cells with unpolarized myosin IIB tend to undergo random migration, whereas cells with polarized myosin IIB exhibit persistent migration. **c** Focal adhesion exhibits either stable or dynamically fluctuating traction, and the FAK/phosphopaxillin/vinculin pathway is essential for high FA traction and for tugging FA traction over a broad range of ECM rigidities and guide durotaxis. **d** Filopodia probe the stiffness of the extracellular matrix by using a myosin II-dependent mechanism. The forces generated in lamellipodia are responsible for mechanosensation by regulating the formation of new adhesions. **e** For cells on stiffness gradient gels, the Golgi–nucleus axis determines the distribution of Golgi microtubules, which in turn regulate focal adhesion turnover. When the Golgi microtubules are disrupted, cells with an unpolarized Golgi and enlarged FAs are unable to migrate against the stiffness gradient. **f** Cells that exhibit maximal traction force on “optimal stiffness” are capable of moving away from rigid environments and toward matrices on which they can generate more traction, thus exhibiting negative durotaxis.

The mechanical microenvironment in vivo varies from 1 Pa to over 100 kPa^17^, and this mechanical cue significantly influences cell proliferation, differentiation, and cell migration^18–20^. In 2000, durotaxis was first reported to describe the migration of cells toward the stiffer end of the extracellular matrix^21^ (Fig. 1a). Since then, studies have demonstrated that various types of both single cells and collectives of cells exhibit durotaxis^22–24^. Moreover, a growing number of studies have reported that durotaxis occurs in vivo^22^. A recent review focused on in vivo durotaxis evaluated the evidence supporting its occurrence in vivo^25^. In 2014, the term negative durotaxis was coined for a novel type of cell migration, which is guided toward softer areas by extracellular stiffness (Fig. 1a). The mechanism of negative durotaxis can be explained by the motor-clutch model. Negative durotaxis is thought to contribute to the metastasis of acral melanoma^23^. Both durotaxis and negative durotaxis were found to be guided by extracellular stiffness. However, the connection between durotaxis and negative durotaxis remains unclear.

In this review, we present the underlying mechanisms of durotaxis, including nonmuscle myosin II activity, focal adhesion signaling, the probing of extracellular stiffness by lamellipodia or filopodia, and focal adhesion dynamics regulated by Golgi-derived microtubules. By comparing the mechanisms of durotaxis and negative durotaxis, we highlight the connections between them and propose ways to switch from one to the other. Furthermore, we discuss the impediments encountered during the investigation of durotaxis and negative durotaxis, particularly those related to the generation of stiffness gradient substrates.

## Mechanisms of durotaxis and negative durotaxis

Cell polarization is crucial for directed cell migration, and myosin II plays a vital role in this process^26^. Studies have shown that nonmuscle myosin II appears to be unpolarized when located on a soft matrix or within soft tissue^27^ (Fig. 1b). However, when cells migrate from softer to stiffer matrices, myosin II appears to repolarize, indicating its potential contribution to the process of durotaxis (Fig. 1b). It is important to note that the phosphorylation of myosin IIA S1943 is necessary for the polarization of myosin IIB and subsequent durotaxis. Collective cell migration is a more complicated and efficient phenomenon than single-cell durotaxis. Recent research suggests that the involvement of myosin motors is essential for collective cell durotaxis^24^. Observations have also shown that single MCF-10A cells do not exhibit durotaxis, whereas groups of MCF-10A cells collectively do. A computer model that incorporates focal adhesion, force transmission through cell‒cell junctions, and actin polymerization at the leading edges predicts that the activity of myosin motors and the contractile force contribute to the collective durotaxis of MCF-10A cells. Therefore, mechanosensation by myosin motors plays a crucial role in both individual and collective cell durotaxis.

Filopodia and lamellipodia are actin-rich structures that exist in the leading edge of migrating cells^28^. The polymerization of actin filaments provides a pushing force toward the membrane and generates a protrusion. It has been well documented that the protrusion of filopodia or lamellipodia senses the rigidity of the substrate and regulates the formation of nascent adhesions^29,30^. Cells employ filopodia extensions to investigate the rigidity of the substrate located at a certain distance in front of their leading edge (Fig. 1d). Cdc42 and formins are upstream regulators of filopodia^31^, and the inhibition of Cdc42 and formins hinders actin cytoskeleton turnover, which consequently reduces the detection rate of filopodia. Interestingly, filopodia act as mechanosensors by a myosin II-dependent mechanism^29^. Myosin II is necessary not only for generating probing forces but also for retracting in response to soft substrates. Lamellipodia, which are formed by Arp2/3 complex-derived branched actin, are capable of sensing the rigidity of the extracellular matrix through the regulation of cell spreading and attachment (Fig. 1d). The forces in the lamellipodia, which are independent of myosin II, enable mechanosensation by regulating the formation of new adhesions^30^. For example, on soft substrates, protrusions are not stable due to impaired focal adhesion formation. However, the addition of Mn^2+^ promotes the formation of nascent focal adhesions in the lamellipodia and cell spreading on soft substrates. Although the forces in filopodia and lamellipodia are generated by different mechanisms, they both play important roles in mechanosensation and may regulate several other processes that are sensitive to stiffness.

Focal adhesion within a cell exhibits two states: a stable state and a dynamic state. The rigidity of the extracellular matrix can influence which state is chosen, making it more or less predictable^32^. By characterizing the forces generated by cells on the nanoscale within individual focal adhesions, the Waterman group reported that a complex pathway involving FAK, phosphopaxillin, and vinculin regulates the dynamic fluctuating traction of focal adhesion, causing pulling forces on the ECM^33^. This allows the focal adhesion to sense the rigidity of the ECM and guide durotaxis (Fig. 1c). Moreover, paxillin phosphoregulation and the interaction between paxillin and vinculin limit the range of extracellular matrix (ECM) rigidities to which cells can respond. The conclusion drawn is that tugging traction dynamics within focal adhesion slows random migration and promotes durotaxis.

Microtubules, one of the most important cytoskeletal structures in the cell, play important roles in cargo transportation, force generation, and cell migration^34^. Golgi-associated microtubules are driven independently of MTOC, and CLASPs and AKAP450 are two critical nucleators^35,36^. Recently, it has been reported that Golgi-associated microtubules regulate focal adhesion turnover at the leading edge through the interaction between focal adhesion and the plus end of Golgi microtubules, ultimately regulating durotaxis^37^ (Fig. 1e). Furthermore, the Golgi apparatus can be influenced by external mechanical stimuli, and the alignment of the Golgi-nucleus axis corresponds with the stiffness gradient during durotaxis. Thus far, it remains unclear why microtubules induced by MTOC do not participate in regulating focal adhesion dynamics and therefore do not regulate durotaxis.

The mechanism of negative durotaxis is currently not well researched. A frequently implemented mathematical model for negative durotaxis is the motor-clutch model, which is composed of actin filaments, myosin II (motors) and integrin-mediated focal adhesion (clutches)^38^. Simply put, the polymerization of actin filaments pushes the membrane forward, while the force generated by myosin II pulls actin filaments away from the cell edge and generates retrograde F-actin flow. This retrograde flow can be mitigated by focal adhesion and can bias cell migration toward higher-adhesion microenvironments. The motor-clutch model predicts that cells exhibit biphasic traction forces within a specific stiffness range, and cells generate maximal contraction at the “optimal stiffness”^38,39^. Moreover, the motor-clutch model demonstrates distinct regimes: at high substrate stiffness, clutches undergo frictional slippage, while at low substrate stiffness, clutches experience load-and-fail (Fig. 1f). At the optimal stiffness, clutches are fully extended, exerting maximum resistance on the motors^40^. This implies that cells tend to move away from stiff environments toward matrices on which they can generate more traction, showing negative durotaxis. In contrast, cells on a substrate that is softer than the “optimal stiffness” tend to migrate toward the “optimal stiffness” and thus show durotaxis. It has been reported that U-251MGs, neurons, and B16 cells show maximal contraction on the optimal stiffness and display negative durotaxis^23,38,39^, and this phenomenon can be explained by the motor-clutch model. Although the motor-clutch model is one of the mechanisms of negative durotaxis, further investigation is needed to understand the signaling pathway involved in this process.

In summary, it has been shown that myosin II or structures dependent on myosin II, such as filopodia, as well as focal adhesion signaling or structures regulating focal adhesion dynamics, such as Golgi-driven microtubules, contribute to durotaxis. Additionally, the motor-clutch model contributes to negative durotaxis. Despite numerous studies that have focused on durotaxis and negative durotaxis, the understanding of their mechanisms remains elusive. However, with the development of new technologies and equipment, we are increasingly close to unraveling the mechanisms underlying durotaxis and negative durotaxis.

## The principles of cell migration directed by extracellular matrix stiffness

**Fig. 2:**
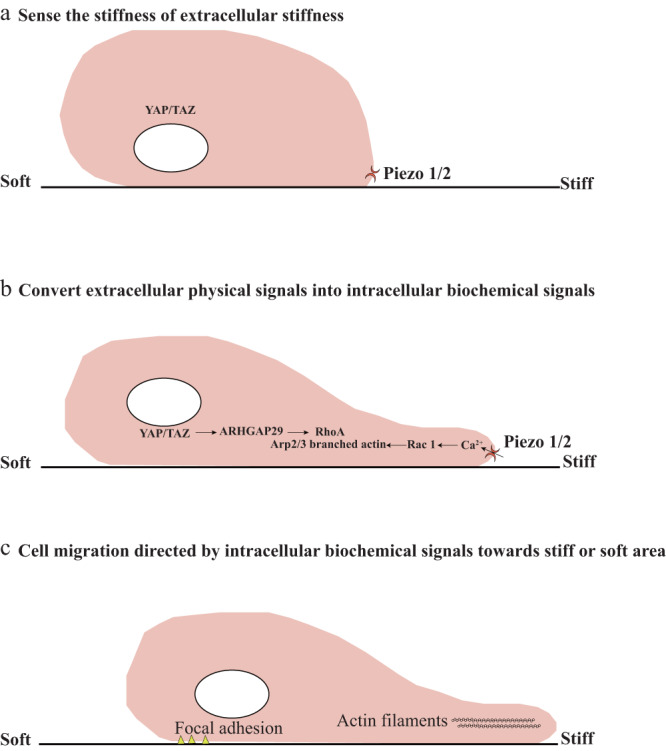
The principles of cell migration directed by extracellular matrix stiffness. **a** Cells on stiffness gradient gels sense the stiffness of the extracellular matrix through mechanosensors, such as YAP/TAZ, nucleus, and piezo1/2. **b** After sensing the stiffness of the extracellular substrate, cells convert extracellular physical signals into intracellular biochemical signals. YAP/TAZ localization inhibits myosin II activity through the ARHGAP29-RhoA signaling pathway. Calcineurin activates Rac1 and promotes the formation of lamellipodia. **c** The intracellular biochemical signal gradient directs cell migration toward the stiff end or the soft end.

According to the mechanisms of durotaxis and negative durotaxis, we can conclude that the principles of cell migration directed by extracellular stiffness include the ability to sense the stiffness of the extracellular matrix, the ability to convert physical signals into biochemical signals, and migration directed by intracellular biochemical signals toward stiff or soft areas (Fig. 2).

### Sensing the stiffness of the extracellular matrix by mechanosensors

There are various mechanosensors in the cell that allow the cell to sense the stiffness of the extracellular matrix. These sensors include YAP/TAZ, Piezo 1/2, and others^41–43^. YAP/TAZ function as downstream effectors of the Hippo signaling pathway and are transported into the nucleus to act as transcriptional coactivators^44,45^. Extracellular matrix stiffness regulates the activity and localization of YAP/TAZ^46^. For example, a stiff substrate promotes the transport of YAP/TAZ into the nucleus, while a soft substrate promotes cytoplasmic localization. The nuclear localization of YAP/TAZ promotes the expression of downstream genes and leads to cell proliferation^45,47^. The expression of actin cytoskeleton regulators, such as Rac1, phosphoinositides, and PI3K, plays a critical role in the regulation of cell migration.

In addition to YAP/TAZ, the nucleus can also function as a vital mechanosensor^48^. Extracellular forces can be transmitted into the nucleus through the LINC complex, resulting in chromatin reorganization and modulation of gene expression^49^. Knocking out the LINC complex disturbs the connection between the actin cytoskeleton and the nucleus, inhibiting the mechanosensation of the nucleus. Piezo 1/2 are mechanically activated ion channels involved in sensing forces in various cells^50,51^. Piezo1/2 senses mechanical forces applied externally at the plasma membrane with high sensitivity, resulting in cation-selective transmembrane transport.

### Convert extracellular physical signals into intracellular biochemical signals

After sensing extracellular matrix stiffness through mechanosensors, cells convert extracellular physical signals into intracellular biochemical signals. For example, a stiff substrate promotes YAP/TAZ localization, which in turn inhibits the activity of RhoA through the overexpression of ARHGAP29^52^. This inhibition of RhoA decreases the activity of myosin II^53^, thus regulating both durotaxis and negative durotaxis. Forces that are transmitted into the nucleus result in chromosome reorganization and transcription modulation^54^. The different expression levels of migration-related proteins regulate durotaxis and negative durotaxis. For example, the expression levels of small GTPases such as Rac1 and RhoA are directly related to cell migration. Moreover, the expression levels of focal adhesion components or signaling related to focal adhesion regulates both durotaxis and negative durotaxis. An expression or activity change in Piezo1/2 transmits signals of fluid shear stress and extracellular matrix stiffness that activate Ca^2+^ influx, stimulating calcineurin^55,56^. This subsequently triggers the concerted activation of the transcription factors NFATc1, YAP1, and β-catenin, inducing their dephosphorylation and promoting the formation of the NFAT/YAP1/β-catenin complex. Moreover, calcium has the ability to activate Rac1 and regulate the direction of cell migration through the formation of lamellipodia.

### Cell migration directed by intracellular biochemical signals toward stiff or soft areas

After the generation of intracellular spatial and temporal gradients of biochemical signals through mechanosensors, cells generate polarity under the regulation of these signals^57^. Additionally, intracellular spatial and temporal gradients of biochemical signals regulate the turnover of the actin cytoskeleton, myosin II activity, and focal adhesion dynamics^58–60^. This ultimately leads to actin polymerization and focal adhesion formation or disassembly, which aligns with the stiffness gradient. Finally, cells are directed to migrate toward either the soft or stiff end under the regulation of intracellular biochemical signals.

Although cells exhibit durotaxis or negative durotaxis according to the three principles, there remains uncertainty regarding the pathways involved in durotaxis and negative durotaxis. Moreover, despite the evidence suggesting that directional migration in vivo is coordinated by extracellular cues, it remains unknown whether cells undergo durotaxis and negative durotaxis in vivo.

## Why do some cell types appear to undergo durotaxis while others undergo negative durotaxis?

The strategy of cell migration included mesenchymal cell migration, amoeboid migration, and collective cell migration. Durotaxis has been extensively studied, and many cells have been found to exhibit positive durotaxis (Table 1). However, only U-251MGs, B16 cells, and neurons are reported to undergo negative durotaxis^23,39,61^. In this context, why do some cell types appear to undergo durotaxis while others undergo negative durotaxis?

**Table 1:** Different cell types undergo durotaxis.

Cell types	Durotaxis modes	Refs.
NIH3T3	Single cell	^29,30,67^
MEFs	Single cell	^33^
MSC	Single cell	^27^
MCF-10A	Collective cell	^24^
MDA-MB231	Single cell	^65,68^
VSMCs	Single cell	^69^
Microglia	Single cell	^70^
C-elegans	Single cell	^71^
PSCs	Single cell	^72^
U87-MG	Single cell	^65^
HT-1080	Single cell	^65^
Xenopus embryo	Collective cell	^22^
MCF7	Single cell	^63^

One possibility is the expression level of the mechanosensor or the activity of the mechanosensor. In MDA-MB-231 cells, YAP/TAZ localizes to the nucleus on stiff substrates and shows cytoplasmic localization on soft substrates^38^. YAP/TAZ function as transcription coactivators and regulate cell contraction through the ARHGAP29-RhoA-Myosin II pathway^52,53^. According to the motor-clutch model, myosin II-mediated contraction and focal adhesion determine the contraction force on substrates of different stiffnesses. This ultimately determines whether a cell undergoes negative or positive durotaxis. The expression level and activity of mechanosensors may play an important role in determining whether a cell undergoes negative or positive durotaxis Box 1.

An alternative possibility is the expression level of adhesion components, such as integrin, talin, vinculin, and paxillin. Among all of these molecules, the adaptor protein talin stands out as a particularly interesting candidate due to its direct linkage of integrins to actin and because it is stretched as cells transmit forces to the ECM. Recently, it has been reported that talin establishes a stiffness threshold, which increases force transmission and triggers force transduction^38^. Additionally, when talin is knocked down, fewer focal adhesions are displayed, and a biphasic contraction force is observed on gradient stiffness substrates^38^. Therefore, the knockdown of adhesion components regulates both cell contraction and adhesion, determining whether a cell undergoes durotaxis or exhibits negative durotaxis.

Box 1 Important developments and future challenges **Important developments**

- With advances in new technologies and methods, we are now able to create substrates with a wide range of stiffness gradients that can be reproduced consistently^24,73^.
- An increasing number of theoretical models are being utilized to elucidate the phenomena of durotaxis and negative durotaxis^39,74^.
- A recent study successfully demonstrated the existence of durotaxis and dynamic stiffness gradients in the neural crest of *Xenopus laevis*^22^.

**Future challenges**

- The mechanism underlying durotaxis and negative durotaxis remains unclear.
- The significance of durotaxis and negative durotaxis in the context of development, homeostasis, and disease remains ambiguous.

## Can a cell change from durotaxis to negative durotaxis or from negative durotaxis to durotaxis?

Both positive and negative durotaxis are forms of cell migration directed by the stiffness of the extracellular matrix. Therefore, it is interesting to examine whether a cell can turn from positive durotaxis to negative durotaxis or vice versa.

The motor-clutch model suggests that cells exhibiting negative durotaxis display biphasic traction forces within the physiological stiffness range. It has been reported that MDA-MB-231 exhibits positive durotaxis, and the knockdown of Talin-1 and Talin-2 switches positive durotaxis to negative durotaxis^38^. Therefore, the upregulation of Talin-1 and Talin-2 may turn negative durotaxis to positive durotaxis.

The expression level or activity of mechanosensors can also regulate focal adhesion dynamics. It has been reported that YAP/TAZ regulate cell mechanics by restricting the maturation of cytoskeletal and focal adhesion, thereby enabling persistent cell motility^52,53^. This suggests that the expression level or activity of YAP/TAZ can determine whether a cell exhibits positive or negative durotaxis.

## What are the impediments in the investigation of durotaxis and negative durotaxis?

There are numerous impediments to investigating durotaxis and negative durotaxis, and one such challenge is the lack of robust experimental approaches to generate stiffness gradients. The earliest method for generating stiffness gradients included pipetting two drops of polyacrylamide solution, each with a different concentration, onto a coverslip, then compressing them together with a removable upper coverslip^21,27,62^. However, the gradient gel produced by this method exhibits a short stiffness range, and the stiffness gradient is nonlinear. Using a grass micropipette, investigators are able to create a stiffness gradient through the physical stretching or deformation of polyacrylamide gel near individual cells^33,63,64^. However, this approach has a low throughput, and it is unclear what kind of stiffness gradient is generated by this method. Another approach involves combining a moving mask with photopolymerization gel to create a stiffness gradient^24,37,65^. This technique requires a precisely controlled moving mask and a stable UV lamp, and it is currently the most frequently used method.

The other obstacles in the investigation of durotaxis and negative durotaxis include the lack of a robust way to exclude other stimuli apart from the mechanical cue. For instance, cells that are cultured on a stiffness gradient could secrete fibronectin, laminin, and collagen, thereby causing remodeling of the extracellular matrix. This remodeling could result in haptotaxis instead of durotaxis or negative durotaxis.

Other obstacles include a lack of adequate methods to measure tissue stiffness and track the migration of cells in living animals^66^. To gain a more comprehensive understanding of durotaxis and negative durotaxis, we must overcome these challenges.

## Concluding remarks

Both durotaxis and negative durotaxis are characterized by cell migration directed by the stiffness of the extracellular matrix. Despite the distinct mechanisms underlying these two processes, there is a close connection between them. It is highly likely for cells to switch from durotaxis to negative durotaxis or vice versa. With the development of technology for generating stiffness gradient gels, the mechanisms and in vivo functions of both durotaxis and negative durotaxis will be elucidated. Furthermore, it will be crucial to identify the key regulators in the signaling pathways of both durotaxis and negative durotaxis. This will enable the development of inhibitors that specifically target these regulators and effectively hinder the processes of durotaxis and negative durotaxis. This approach will provide novel strategies for treating various diseases caused by durotaxis and negative durotaxis.

### Reporting summary

Further information on research design is available in the Nature Portfolio Reporting Summary linked to this article.

### Supplementary information

Reporting Summary
